# Neuroimmune cell interactions and chronic infections in oral cancers

**DOI:** 10.3389/fmed.2024.1432398

**Published:** 2024-07-10

**Authors:** Nisha J. D’Silva, Pushpa Pandiyan

**Affiliations:** ^1^Department of Periodontics and Oral Medicine, School of Dentistry, University of Michigan, Ann Arbor, MI, United States; ^2^Department of Pathology, Medical School, University of Michigan, Ann Arbor, MI, United States; ^3^Rogel Cancer Center, University of Michigan, Ann Arbor, MI, United States; ^4^Department of Biological Sciences, School of Dental Medicine, Case Western Reserve University, Cleveland, OH, United States; ^5^Department of Pathology, Case Western Reserve University, Cleveland, OH, United States; ^6^Center for AIDS Research, Case Western Reserve University, Cleveland, OH, United States; ^7^Case Comprehensive Cancer Center, School of Medicine, Case Western Reserve University, Cleveland, OH, United States

**Keywords:** neuroimmune, chronic, HIV, *Candida*, T_reg_, perineural invasion, PNI, immune suppression

## Abstract

Inflammation is a process that is associated with the activation of distal immunosuppressive pathways that have evolved to restore homeostasis and prevent excessive tissue destruction. However, long-term immunosuppression resulting from systemic and local inflammation that may stem from dysbiosis, infections, or aging poses a higher risk for cancers. Cancer incidence and progression dramatically increase with chronic infections including HIV infection. Thus, studies on pro-tumorigenic effects of microbial stimulants from resident microbiota and infections in the context of inflammation are needed and underway. Here, we discuss chronic infections and potential neuro-immune interactions that could establish immunomodulatory programs permissive for tumor growth and progression.

## Introduction

Chronic infections have been identified as a significant risk factor for the development of various types of cancer. *Helicobacter pylori*, human papillomavirus (HPV), hepatitis B virus (HBV), hepatitis C virus(HCV), and Epstein–Barr virus(EBV) play prominent roles (1). Other infections such as *Candida* are also associated with cancers (2). Infectious agents and inflammation modulate a broad range of host immune responses, which in turn may promote carcinogenesis and progression (3). However, mechanisms related to infection-mediated immunomodulation in tumor development have not been completely understood. Local inflammatory signals in tissues provide a positive feedback loop to nerve fibers, glial, and immune cells which dynamically reciprocate in a recurrent fashion in the tumor microenvironment (4, 5). Here we discuss some of these interactions in intra-tumoral immunosuppressive milieu and cellular crosstalk at the neural-immune interface that may contribute to oral cancer initiation, progression, and metastasis.

## CD8 exhaustion and CD4^+^CD25^+^FOXP3^+^ regulatory cells (T_regs_) – at the crossroads of immune-homeostasis, chronic infections, and tumor evasion

Chronic and persistent inflammatory stimuli (due to ongoing HIV replication, microbial translocation, or co-infections) have been shown to stimulate the expression of pro-inflammatory cytokines in people living with HIV (PLWH) (6–11). Accumulating evidence suggests that HIV may lead, at least in part, to an accelerated aging phenotype in immune cells and immunomodulatory/inflammaging phenotype (12–17). With regards to the oral mucosa, opportunistic infections (18) and altered oral microbiome/mycobiome profiles (19–22) are also important features of oral inflammation in PLWH under treatment (23–26). Local and systemic inflammation in PLWH are linked to a wide range of co-morbidities, the most significant being increased propensity to malignancy (27–30). Some oral malignancies in PLWH are linked to HPV, EBV, and Kaposi’s sarcoma herpesvirus, as well as the HIV itself (31). However, the immune mechanisms of increased propensity to Head and neck cancers (HNC) in PLWH are not fully understood. HNC are the sixth most common cancers, accounting for 450,000 GLOBOCAN estimated deaths each year (32, 33). Over two-thirds of all new cancers are diagnosed among adults aged ≥60 years. Oral squamous cell carcinoma (OSCC) are aggressive tumors constituting ~90% of all oral cancers, with a global incidence of ~350,000 new cases and 177,000 deaths annually. The treatment of OSCC mainly includes surgery, radiotherapy, and chemotherapy. The prognosis of OSCC is poor due to tumor recurrence (50%) or lymph node metastasis within 3 years, and the 5-year survival rate is ~50% (34, 35). Although our understanding of underlying oncogenic processes of OSCC is evolving and has led to Epidermal Growth Factor Receptor (EGFR) and PD-1 targeted therapies, major roadblocks exist. Several cancers, including a large proportion of oral cancers, do not respond to immune checkpoint inhibitors (36–40). Varied treatment efficacy between patients, inadvertent side effects, overtreatment, worsening prognosis, and increasing treatment costs contribute to treatment challenges. Therefore, there has been great interest in understanding the mechanisms that govern immunosuppression in cancer including the contributions of components of the tumor immune microenvironment (TIME).

Due to their crucial role in the anti-tumoral immune response, CD8^+^ T lymphocytes and their dysfunction have been a major focus of attention. In the context of chronic infections, inflammation, or cancer progression, persisting antigen stimulation of CD8^+^ T cells drives progressive loss of functionality and eventual deletion instead of memory formation (41, 42). The cells in TIME are known to release cytokines to engage checkpoints on immune cells, induce an increase in exhausted cytotoxic T cells, thereby disrupting the anti-tumoral immune response. Also, unceasing antigen stimulation in tumors profoundly alters T cell differentiation trajectories, leading to CD8^+^ precursors of exhausted T cells (T_PEX_ cells), which share several common features with those in chronic infections (43). Exhausted CD8 T cells may comprise heterogeneous cell populations expressing a multitude of exhaustion markers such as TOX, PD1 and lymphocyte-activation gene 3 protein (LAG3) and memory cell markers including T cell factor 1 (TCF1), B cell lymphoma 6 protein (BCL6), inhibitor of DNA binding 3 (ID3) and SLAMF6 (also known as LY108), C–C chemokine receptor type 7 (CCR7), CD62L, CD127, CD69 and eomesodermin (EOMES) (44–46) and are present in both lymphoid and non-lymphoid tissues. Apart from T_PEX_ cells, effector-like, tumor-reactive, exhausted CD8^+^ T cells (TEX cells) that are distinct from resting memory T cells are also found in TIME (47, 48). These subsets undergo gradual exhaustion in the face of persisting antigen stimulation giving rise to effector-like cytolytic exhausted T cells (T_EEF_ cells), transient or intermediate differentiation state exhausted T cells (T_INT_ cells) with antitumoral functions, as well as terminally exhausted T cells (T_TEX_ cells) along this trajectory. It will be of considerable interest to determine the functional relevance of these stem cell–like and exhausted CD8 T cells and factors that may drive their proliferation or exhaustion in the context of chronic infections and OSCC.

FOXP3^+^T_regs_ are central to immune homeostasis but have been implicated in cancer immune evasion and angiogenesis (49–53). These cells, along with tumor-associated macrophages (TAM), and myeloid-derived suppressive cells (MDSC) accumulate in tumors and contribute to poor immunologic response against the tumor (54, 55). T_regs_ display a broad degree of functional heterogeneity and phenotypic plasticity within tissues and tumors Indeed we (17, 27, 56–58) and others have shown distinct populations of T_regs_ namely, T-bet^+^FOXP3^+^cells (that may be dysfunctional; T_regDys_), ROR-γt^+^FOXP3^+^ (T_reg17_) cells, and PD-1^+^FOXP3^+^ cells (16, 17, 59), whose functions are significantly altered by microbiome, IL-6, and IL-1β in an mTOR dependent manner in oral mucosa (58). In solid tumors of nonlymphoid origin, T_regs_ may constitute 30–45% of CD4^+^ T cells, depending on the tumor type (55, 60), and can also hinder the success of α-PD1 cancer immunotherapy (61). Having a high T_reg_ infiltration and highest T_reg_/CD8^+^ T cell ratio among all cancers, OSCCs are poised to benefit from T_reg_-targeted approaches (60). Intra-tumoral FOXP3 + T_reg_: CD8 ratio is associated with poor prognosis and survival in human OSCC (62) and is linked to pro-tumorigenic functions of T_regs_ (55). Besides being recruited into tumors via chemotaxis, FOXP3^+^ T_regs_ can be induced *in situ* in tumors by mediators released from tumor cells, TAM and MDSC (63). Increased accrual of T_regs_ is also observed in aging (age > 60) oral mucosa compared to younger mucosa (58, 64). In a 4-Nitroquinoline 1-oxide (4-NQO) mouse model of oral carcinogenesis, *Candida albicans* infection and zymosan exacerbate and accelerate dysplasia and hyperplasia demonstrating the role of fungal ligands in exacerbating tumor growth and progression. Our prior studies have established the requirement of TGF-β1 and microbiome in T_reg_ cell induction and viability during *Candida* infection (59, 65–68). Several studies have suggested a link between oral fungi and the development of OSCC (2, 55, 69). Yet, the underlying molecular mechanisms of OSCC initiation and progression are unknown. A combination of inflammaging and impaired immunity contributes to increased susceptibility to infections and cancer in elderly individuals. *Candida* infection is effectively cleared in young mice but causes oral inflammation in aged mice (58). Immunopathology involved the loss of anti-inflammatory function by IL-1β dependent T_reg_17, but an accumulation of IL-6 and T_regDys_ in aged oral mucosa (58). These data suggest that aging may lead to loss of homeostatic mechanisms that maintain *Candida* in a non-inflammatory commensal state. Also, aged mice show increased fungal abundance and early filtration of T_regs_ and MDSC cells than young mice during oral tumorigenesis (55). Tumors in aged animals further show higher PD-1 expression (exhaustion marker) in CD8^+^ T cells coinciding with accelerated incidence of dysplasia, hyperplasia, and OSCC development when compared to younger mice. Elevated resident fungal abundance in saliva implies the role of resident mycobiome dysbiosis in promoting immune dysfunction and tumorigenesis, although the cause versus consequence effect of the mycobiome is unknown. In summary, while evidence point to a link between inflammaging and immunomodulation mechanisms, the process by which mucosal cells are precisely poised for tumor growth in different contexts needs further exploration.

Fungal recognition receptors such as TLR-2 and dectin-1 are expressed by myeloid dendritic cells, monocytes, and macrophages. Dectin-1 binds specifically to β-1,3 glucans in fungi, as well as to endogenous galectins and annexins on apoptotic cells (70, 71). It has a C-type lectin-like carbohydrate recognition domain, whose stimulation leads to phosphorylation of Syk (p-SYK) and IL-1β secretion. Fungi can also induce inflammasome activation by signaling through MyD88/NF-κB and Caspase recruitment domain-containing protein-9/SYK pathways and engage pyrin domain-containing protein 3 (NLRP3), which activates the protease caspase-1 in infected macrophages and other immune cells (72, 73). IL-1β is a typical cancer-inflammation-associated cytokine up-regulated in saliva derived from OSCC patients and is linked to poor prognosis for esophageal cancer (74). HIV can also activate inflammasome pathway and IL-1β secretion, which are linked to AKT activation, T cell dysfunction and T_reg_ enrichment seen in oral mucosa of PLWH (16). *Candida* can further potentiate inflammasome pathway in the context of HIV (15, 16). IL-1β is also involved in maintaining immunomodulatory Foxp3^+^ROR-γt^+^ (T_reg17_) cells (56–58, 68, 74), which contribute to mucosal homeostasis, tumor immune evasion and autoimmunity control (75). Inflammasome activation can significantly alter the population size and functions of immune cells (76, 77), and are linked to tumor initiation and development (78). NLRP3 is also implicated in promoting Th1 responses and anti-tumor immune functions (79). Inflammasome activation and metabolic pathways are intricately connected and regulate each other through feed-back loop mechanisms. Activated caspase-1 can mediate multiple processes including (1) release of IL-1β, (2) pyroptosis (80), (3) mitochondrial damage (81), (4) cleavage of glycolytic enzymes (82) causing alterations in glycolytic metabolites (83), and (5) degradation of innate immune sensor proteins (84). For example, succinate, an intermediate of the tricarboxylic acid (TCA) cycle can activate NLRP3 through HIF-1α stabilization and reactive oxygen species production (85). Similarly, K^+^-depletion/efflux-induced canonical NLRP3 response is associated with increased glycolytic flux, which is dependent on the AKT/PI3-K/ mammalian target of rapamycin (mTOR) pathway, and upregulation hexokinase 1 the primary glycolytic enzyme (86). Therefore, immunometabolism and inflammasome pathways play pivotal roles in integrating growth signals and functions in T cells including T_regs_ and govern tumor permissive pathways (87–93). Understanding them will pave the way to new combinatorial strategies in the face of resistance to PD-1 immunotherapy, leading to improved patient outcomes.

## Interactions between immune cells and neural cells in inflammation

The oral mucosa is richly innervated with sensory afferents for physiological sensory perception (94, 95). Innervation for the oral mucosa is from the maxillary and mandibular branches of the trigeminal nerves, facial, glossopharyngeal, vagus, and hypoglossal nerves; and by the spinal accessory nerve (96–98). The tongue receives additional sensory innervation from the glossopharyngeal nerve, and the chorda tympani branch of the facial nerve (97, 99). Bidirectional signals between tissue-resident immune cells and nerve fiber terminals form an integrated network coordinating and modulating antimicrobial immunity, inflammation and pain signals during infection, and tissue homeostasis (100–102). For example, microglia were found to cross-present antigen after acquisition from adjacent olfactory sensory neurons and provide a front-line defense against a neuroinvasive nasal viral infection (103). Residential macrophages play a homeostatic role in the control of tissue innervation of brown-adipose tissue (104). Tissue-residential T_regs_ promote myelin regeneration upon damage of the central nervous system mediated by CCN2 (105). Infective agents, damaged host cells, and activated immune cells may initiate inflammatory signals in the nerve fiber environment (106). Such inflammatory chemical signals interacting with sensory nerve fiber terminals strongly associate with pain (107–109). It occurs via the synthesis and release of inflammatory mediators such as prostaglandin (PG) and interactions with neurotransmitters and their receptors (107, 110). Arachidonic acid is a key lipid mediator driving pain and inflammatory responses (111, 112) and is metabolized by cyclooxygenase and 5-lipoxygenase, resulting in the synthesis of PG and leukotrienes. This pathway is involved in the release of PGE2, IL-1β and ATP (113) and neuronal nociceptor activation (113–115). Thus, emerging evidence demonstrate how tissue resident immune cells, mucosal and submucosal glial cells, and neurons are actively involved in tissue homeostasis, inflammation, and pain pathophysiology (114–116). Even stress-susceptible cellular and behavioral phenotypes are causally mediated by dectin-1, an innate immune receptor expressed in intestinal γδ T cells (117). Nerves also have an extensive and well recognized role in immune regulation via neurotransmitter neuropeptides such as calcitonin gene-related peptide (CGRP) and substance P (118). Glial cells surrounding trigeminal neurons (119, 120), also produce PGE2 during tissue injury or inflammation and may regulate the sensory neuronal function both in paracrine and autocrine manners (121–123), involving CGRP and SP (124–126). CGRP is a crucial neurotransmitter of sensory neurons innervating the mucosa (127, 128), although it’s modulatory role in oral mucosal immunity needs new exploration.

## Interactions between immune cells and neural cells in tumor progression

In the last two decades, cancer neuroscience has revealed the significant role of nerves in cancer progression (129). Since nerves regulate tumor progression and immunity, there is emerging interest in the nerve-immune-cancer axis. Studies in cutaneous cancers suggest that the increased innervation and damage of nerves in cancer can promote adverse outcomes by favoring pro-tumoral immunity. Nerves in oral squamous cell carcinomas exhibit damage (97, 130, 131). Moreover, increased nerve density is associated with poor outcomes (132). Cytotoxic CD8+ T lymphocytes were recently shown to express RAMP1 (receptor activity-modifying protein 1), the receptor for the neuropeptide CGRP, which is produced by transcription of the Calca gene in mice (133). Balood et al. showed that nociceptors release CGRP that induce RAMP1 on CD8^+^ T cells thereby leading to functional exhaustion (133). These CD8^+^ T cells exhibited concurrent expression of exhaustion markers such as PD-1^+^LAG3^+^TIM3^+^ and suppression of effector functions, resulting in tumor progression. Importantly, ablation of the nociceptor neurons inhibited tumor growth, which was reversed by intra-tumoral injection of CGRP. The importance of these findings lies in the ability to sensitize tumors to immunotherapy by interrupting the cancer-neuro-immune axis. Perineural Invasion (PNI) is another phenomenon that is highly correlated with poor prognosis, increased likelihood of metastasis, higher recurrence rates, node involvement, and decreased survival in OSCC (97, 130, 131, 134, 135). Although it is a high-risk adverse feature in OSCC, there are currently no treatments targeting PNI, which is important route of tumor dissemination in OSCC. It is seen in most of OSCC and provides a challenge to complete resection due to neural extension away from the primary tumor that is missed during surgical margin evaluation. PNI requires crosstalk between multiple cells, paracrine signaling, and direct matrix remodeling in the perineural niche (136–138). TIME-produced mediators activate and sensitize primary afferent neurons, contributing to inflammation, peripheral nerve injury, and sensitization that also underlie pain associated with PNI (128, 139–141). The heterogeneity in levels of neurotropism and the predictive value of nerve-tumor distance for survival among N_0_ patients emphasizes the need for mechanistic studies and characterization of interactions between immune cells, nerves, and cancer cells in TIME. Recent findings in cutaneous squamous cell carcinoma lend support to the importance of nerve-immune interactions in tumor resistance to immunotherapy (142). Non responders to anti-PD-1 therapy are more prone to nerve damage and immunosuppression. In mice, denervation enhanced tumor sensitivity to anti-PD-1 therapies. In a syngeneic orthotopic mouse model of oral squamous cell carcinoma, tumors in Calca knockout mice were smaller than in wild type mice, and had an increased anti-tumor immune response, including CD8^+^ T cells and CD4^+^ T cells (143). In a subsequent study, surgical denervation of the lingual nerve in mice, inhibited tumor growth, enhanced cytotoxic activity of CD8^+^ T cells and improved response to anti-PD1 immunotherapy (144). Findings from these oral cancer studies were corroborated in a recent *in vitro* and *in vivo* study (145).

**Figure 1:**
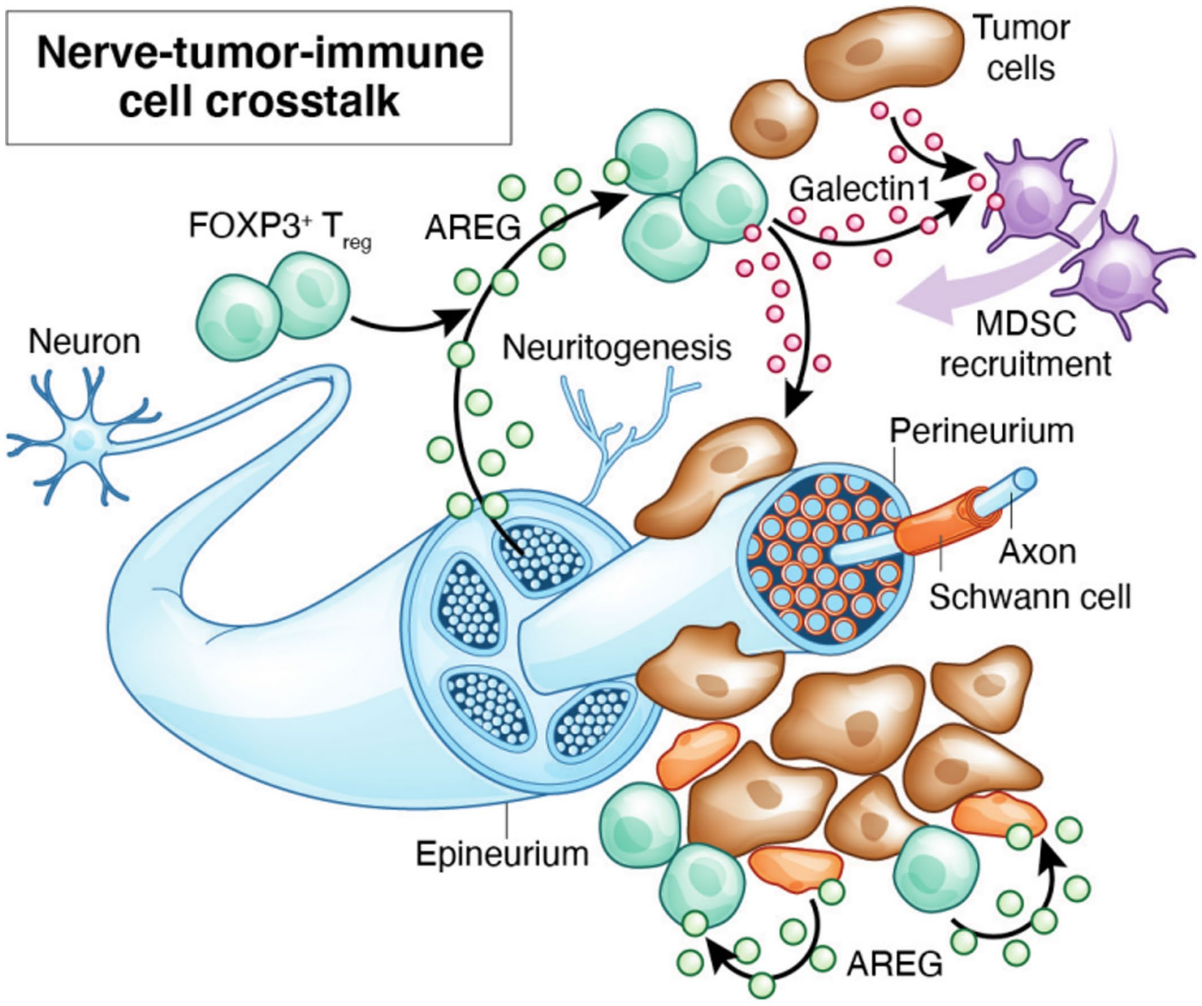
By releasing inflammatory mediators, tissue resident glial cells such as mucosal glia and Schwann cells and T_regs_ may modulate the synthesis and release of neurotransmitters and their receptors. Local cytokines may provide a positive feedback loop through which these cells interact in a reciprocal and recurrent fashion to induce neuropathological and immunosuppressive signals in PNI. These signals may be amplified by infections.

Neuregulins (NRGs) and neurotrophic factors such as nerve growth factor (NGF) are expressed by leukocytes and can be involved in neuro-immune crosstalk. Amphiregulin (AREG) is a glycoprotein that was originally named Schwannoma-derived Growth Factor (SDGF) and is known to be neurotrophic (146). Schwann cell-derived AREG enhances nerve regeneration during peripheral nerve injury. AREG triggers EGFR signaling activating MAPK/ERK, PI3K/AKT, mTOR and STAT pathways in leukocytes (147). It is vital for tissue repair and the suppression of inflammation but is overexpressed in cancers (100, 148). AREG is also upregulated in T_regs_ in oral mucosa under chronic HIV infection, and may enhance their proliferation (16). GAL-1 is present in cytosolic compartments and as secreted form. It is known to promote cell–cell and cell-matrix communications and interact with glycoconjugates in TIME (149). GAL-1 overexpression is observed in the lymphocyte populations adjacent to areas of perineural spread and is associated with poor disease-free survival and overall survival (150). GAL-1 is also known to be upregulated in FOXP3^+^ T_regs_ supporting their differentiation, expansion, recruitment, and immunosuppressive potential (151). However, much work remains to define precise cellular sources of these proteins and their functions at the neuroimmune interface during PNI (Figure 1).

Schwann cells (SC) are glial-type cells that nurture neurons during development, promote myelination of mature peripheral nerves, and play a crucial role in neural regeneration and mediate bidirectional interactions between inflammation and pain (136, 137, 139, 152, 153). An increase in non-myelinating SC, like those responding to nerve injury, is observed in close proximity to pancreatic cancer cells in patient specimens and correlates with tumor invasion and diminished survival in patients (136–138). While GFAP^+^ and S100β^+^ glial cells can be found in oral mucosa (154, 155), it will be crucial to study if these cells support and modulate bidirectional neuronal and immune signaling in TIME (141, 156). Tumor cells can activate c-Jun–dependent reprogramming and kynurenine metabolism changes inducing them into non-myelinating/repair SC, which are involved in tumorigenesis (157). Except these recent reports in pancreatic cancer, the role of SC remains largely unexplored but could be an important cell-type explaining the heterogeneity of OSCC and amenable to therapeutic targeting in OSCC. Taken together, these studies suggest that the nerves and nerve-glia-immune interactions significantly impact cancer progression and could have a crucial role in the outcome of immunotherapy in oral cancer (Table 1).

**Table 1:** Cellular interactions in neuro-immune niche.

Authors	Neuroimmune interactions	Function
Sun, C., et al	Tumor-associated nonmyelinating Schwann cell-expressed PVT1 promotes pancreatic cancer kynurenine pathway and tumor immune exclusion.	Pro-tumorigenic
Ji, R.R., et al	Pain regulation by non-neuronal cells and inflammation.	Pain
Huang, T., et al	Schwann Cell-Derived CCL2 Promotes the Perineural Invasion of Cervical Cancer.	Pro-tumorigenic
Chen, S., et al	Schwann cell-derived amphiregulin enhances nerve regeneration via supporting the proliferation and migration of Schwann cells and the elongation of axons.	
Darragh, L.B., et al.	Sensory nerve release of CGRP increases tumor growth in HNSCC by suppressing TILs.	Pro-tumorigenic
Tao, Z.Y., et al.	Lingual Denervation Improves the Efficacy of Anti-PD-1 Immunotherapy in Oral Squamous Cell Carcinomas by Downregulating TGFbeta Signaling.	Pro-tumorigenic
Deborde, S., et al.	Reprogrammed Schwann Cells Organize into Dynamic Tracks that Promote Pancreatic Cancer Invasion.	Pro-tumorigenic
Schmitd, L.B., et al.	Spatial and Transcriptomic Analysis of Perineural Invasion in Oral Cancer.	Pro-tumorigenic
Balood, M., et al.	Nociceptor neurons affect cancer immunosurveillance.	Pro-tumorigenic
Perez-Pacheco, C., et al.	Increased Nerve Density Adversely Affects Outcome in Oral Cancer.	Pro-tumorigenic
Scheff, N.N., et al.	Tumor necrosis factor alpha secreted from oral squamous cell carcinoma contributes to cancer pain and associated inflammation.	Inflammation
Wang, H., et al.	Enteric neuroimmune interactions coordinate intestinal responses in health and disease.	Barrier immunity
Dombrowski, Y., et al.	Regulatory T cells promote myelin regeneration in the central nervous system.	Immunomodulation
Tsou, A.M., et al.	Neuropeptide regulation of non-redundant ILC2 responses at barrier surfaces.	Barrier immunity
Chiu, I.M., et al.	Bacteria activate sensory neurons that modulate pain and inflammation.	Infection
Talbot, S., et al.	Silencing Nociceptor Neurons Reduces Allergic Airway Inflammation.	Barrier immunity
Moseman, E.A., et al.	T cell engagement of cross-presenting microglia protects the brain from a nasal virus infection.	Barrier immunity
Mailhot, B., et al.	Neuronal interleukin-1 receptors mediate pain in chronic inflammatory diseases.	Inflammation

## Discussion

Cellular dysregulation related to microbiome dysbiosis, infections, and chronic inflammation that could result in malfunction at the neuroimmune interface contributes to initiation and progression of cancers. While infections are associated with tumors, precise mechanisms linking infection responses, immuno-senescence, immunosuppression in tumors are yet to be studied. A new understanding of neurons and glial cells in immune exhaustion and suppression, and tumor development and dissemination of squamous cell cancers are crucial for opening new avenues of investigation leading to better treatment selection and developing new treatment strategies.

## Data availability statement

The original contributions presented in the study are included in the article/supplementary material, further inquiries can be directed to the corresponding author.

## Ethics statement

The studies involving humans were approved by Institutional review board, UH. The studies were conducted in accordance with the local legislation and institutional requirements. The participants provided their written informed consent to participate in this study. The animal study was approved by Institutional Animal Care and Use Committee (IACUC). The study was conducted in accordance with the local legislation and institutional requirements.

## Author contributions

ND'S: Writing – original draft, Writing – review & editing. PP: Conceptualization, Writing – original draft, Writing – review & editing.
